# Impact of thromboplastin reagents on monitoring INR in a patient with triple-positive antiphospholipid syndrome: a case report

**DOI:** 10.3389/fimmu.2025.1591029

**Published:** 2025-07-31

**Authors:** Lóránt Varju, Zsuzsa Bagoly, Éva Ajzner, Rita Orbán-Kálmándi, Anna Zsófia Kádár, Judit Nevelős, Péter Ilonczai

**Affiliations:** ^1^ Department of Hematology, Szabolcs-Szatmár-Bereg County Teaching Hospitals, András Jósa Hospital, Nyíregyháza, Hungary; ^2^ Kálmán Laki Doctorate School, University of Debrecen, Debrecen, Hungary; ^3^ Division of Clinical Laboratory Science, Department of Laboratory Medicine, Faculty of Medicine, University of Debrecen, Debrecen, Hungary; ^4^ Lendület “Momentum” Hemostasis and Stroke Research Group of the Hungarian Academy of Sciences, Debrecen, Hungary; ^5^ Central Laboratory, Szabolcs-Szatmár-Bereg County Teaching Hospitals, András Jósa Hospital, Nyíregyháza, Hungary

**Keywords:** triple-positive antiphospholipid syndrome, recurrent thromboembolism, case report, INR monitoring, antiphospholipid antibodies

## Abstract

**Introduction:**

Antiphospholipid syndrome (APS) is an autoimmune disease characterized by a hypercoagulable state and recurrent thromboembolism (TE). Patients with triple-positive antiphospholipid antibodies (APAs) are at the highest risk of TE. As standard treatment for these patients, oral anticoagulation therapy (OAT) with vitamin K antagonists (VKAs) is widely used, but inaccurate International Normalized Ratio (INR) measurement due to APA interference can complicate monitoring.

**Case:**

Here we report the case of a 19-year-old male patient, with a history of submassive pulmonary embolism at the age of 13. Thrombophilia investigations confirmed type II antithrombin deficiency (Budapest 3 heterozygous) combined with triple-positive APS. He received sustained VKA (warfarin) therapy, but his INR values showed strikingly different results when monitored in two different laboratories (INR 3–4 vs. INR >8 on multiple occasions). Therefore, we aimed to investigate the impact of different thromboplastin reagents on INR values in this triple-positive APS patient receiving VKA therapy. INR measurements were performed using animal-derived (rabbit brain-derived) and recombinant thromboplastins. The effect of purified patient IgG concentrates was examined on INR values using antiphospholipid antibody-negative plasma mixtures. Chromogenic FXa activity (CFXa) was also measured to assess the true anticoagulant effect of VKA.

**Conclusions:**

INR values measured using recombinant thromboplastin reagent were consistently higher and less reliable in high APA-titer conditions compared to rabbit brain-derived reagent. CFXa results were more consistent with INR values obtained using rabbit brain-derived thromboplastin. Rabbit brain-derived thromboplastin, less sensitive to APA interference, provided reliable INR monitoring for this high-risk patient. We recommend choosing thromboplastin reagents without interference to APAs, to optimize OAT monitoring in similar cases of patients with high APA-titers.

## Introduction

Antiphospholipid syndrome (APS) is an autoimmune disease characterized by recurrent thromboembolism (TE) and obstetric complications. The revised Sapporo criteria for APS require at least one clinical criterion (vascular thrombosis and/or pregnancy morbidity) and one laboratory criterion, which can be any of the following: lupus anticoagulant (LA), anticardiolipin (aCL) IgG or IgM, or anti-β2-glycoprotein I (aβ2GPI) IgG or IgM, confirmed on two or more occasions, at least 12 weeks apart (1–3). In the 2023 American College of Rheumatology (ACR)/European Alliance of Associations for Rheumatology (EULAR) APS classification criteria, the type of laboratory parameters remain essentially unchanged compared with the updated Sapporo classification criteria, but aCL and aβ2GPI measurement are restricted to enzyme-linked immunosorbent assays (ELISAs) with moderate and high titer aPL thresholds defined as 40 and 80 Units (4). It must be noted, that the ACR/EULAR classification criteria, having excellent specificity, are meant for participant inclusion in studies and trials to study homogeneous populations of patients, and in general, laboratory detection for APS diagnosis in daily practice remain broader, meant to diagnose each APS patient to optimize their management (3, 4). In APS, thromboses are caused by APA-induced vascular cell activation, inhibition of natural anticoagulants and the fibrinolytic system, and complement activation (2, 5). The triple-positive form of APS presents the highest TE risk, which is characterized by the simultaneous occurrence of LA, ACL and aB2GP1 (6), and is often associated with catastrophic APS and pregnancy loss (7). The risk of recurrent TE is the highest in the triple-positive group of APS patients (8, 9). Secondary TE prevention is the primary goal in APS patients (10, 11). Although direct oral anticoagulants (DOACs) have been investigated, their safety and efficacy in APS are debated, and are not recommended for triple-positive APS (12–14). Instead, vitamin K antagonists (VKAs) remain the cornerstone anticoagulation therapy (15).

INR monitoring in APS patients can be challenging due to LA interference, which affects thromboplastin reagents differently (16, 17). If a TE event occurs despite VKA therapy, the thromboplastin reagent’s LA sensitivity should be reviewed, and confirmatory testing (e.g., FXa amidolytic assays) should be performed (11).

## Case description

We report the case of a 19-year-old male with a history of deep vein thrombosis (DVT) at the age of 12 and submassive pulmonary embolism (PE) at 13 years old. Thrombophilia investigations confirmed type II antithrombin deficiency (Budapest 3 heterozygous) and triple-positive APS. Despite sustained VKA (warfarin) therapy, INR values varied significantly between two laboratories (INR 3–4 vs. INR >8). To investigate these discrepancies, we assessed the impact of different thromboplastin reagents on INR measurements using the patient’s plasma and IgG purified from the patient’s serum.

The presence of LA was confirmed based on the screening and confirmatory tests in accordance with the revised Sapporo recommendations (1). The quantitative determination of aB2GP1 IgG and IgM and ACL IgG and IgM antibodies was performed using the ELISA method (QUANTA Lite, Inova Diagnostics). IgG of the triple-positive APS patient was purified by affinity chromatography (MabTrap Kit, GE Healthcare). The protein content obtained by chromatography was measured using a Multiskan Sky photometer (Pierce BCA Protein Assay Kit, Thermo Scientific). We examined the effect of purified patient IgG concentrates on coagulation screening tests, with special regard to INR. The measurements were also performed on an aPL-negative plasma mixture with therapeutic (2.0-3.5) and above therapeutic (>3.5) range of INR. During the measurements, we used animal-derived (rabbit brain thromboplastin, Dia-PT, ISI: 1.16, Diagon, Budapest, Hungary) and recombinant thromboplastin (Innovin, ISI: 0.91, Siemens Healthcare Diagnostics, Marburg, Germany) thromboplastin reagents. As control tests, measurements were performed with the purified IgG of aPL-negative individuals. The effect of VKA was verified by chromogenic FX activity (CFXa) measurements, using a commercially available amidolytic method (Siemens Healthcare Diagnostics, Marburg, Germany).

The patient’s detailed history includes hospitalization at the age of 10 (in 2006) due to bilateral pneumonia that was treated with antibiotics and healed without further complications. Two years later he was readmitted for deep vein thrombosis (DVT) in his left lower extremity. Doppler ultrasound revealed thrombosis in the left femoral common vein, profound vein, posterior and anterior tibial veins. Laboratory results showed an elevated D-dimer level (1.17 mg FEU/L, reference threshold < 0.5 mg FEU/L). He was treated with therapeutic doses of low-molecular-weight heparin (LMWH), transitioned to warfarin (6 mg daily, corresponding to a target INR of 2-3, that was achieved and typically maintained with an INR range of 1.7-4). The patient was referred for pediatric hematology evaluation to rule out familial and/or acquired thrombophilia.

At age 13 (February 2009), the patient was hospitalized again due to weakness and exertional dyspnea. Laboratory findings included an INR of 3.36 (using rabbit brain-derived thromboplastin, Dia-PT, ISI: 1.16, Diagon, Budapest, Hungary), APTT of 73 seconds (reference range APTT: 26–36 sec) and a D-dimer level of 0.79 mg FEU/L. A chest X-ray revealed cardiomegaly, while a cardiology examination showed right heart strain, incomplete right bundle branch block, and pulmonary hypertension. Chest computed tomography (CT) identified calcification in the right main pulmonary artery, raising suspicion of thrombus presence. Pulmonary scintigraphy confirmed pulmonary embolism in the affected region. Although thrombophilia testing had been previously suggested, it was only conducted at this point. Laboratory results confirmed lupus anticoagulant (LA) positivity and type II antithrombin deficiency (Budapest 3 heterozygous).

The patient was referred to the National Institute of Cardiology (György Gottsegen Institute, Budapest, Hungary) for consideration of therapeutic embolectomy. Due to the complexity of the case, a consultation was arranged with an international center (Allgemeine Krankenhaus, Vienna, Austria) to perform the procedure. One month later, the patient underwent a successful thrombectomy in Vienna. However, in the postoperative period, a subtotal thromboembolism developed, necessitating a second thrombectomy within 48 hours. This time, antithrombin replacement and low-dose methylprednisolone therapy were administered. The patient’s oral anticoagulation regimen was switched from warfarin to acenocoumarol.

**Figure 1:**
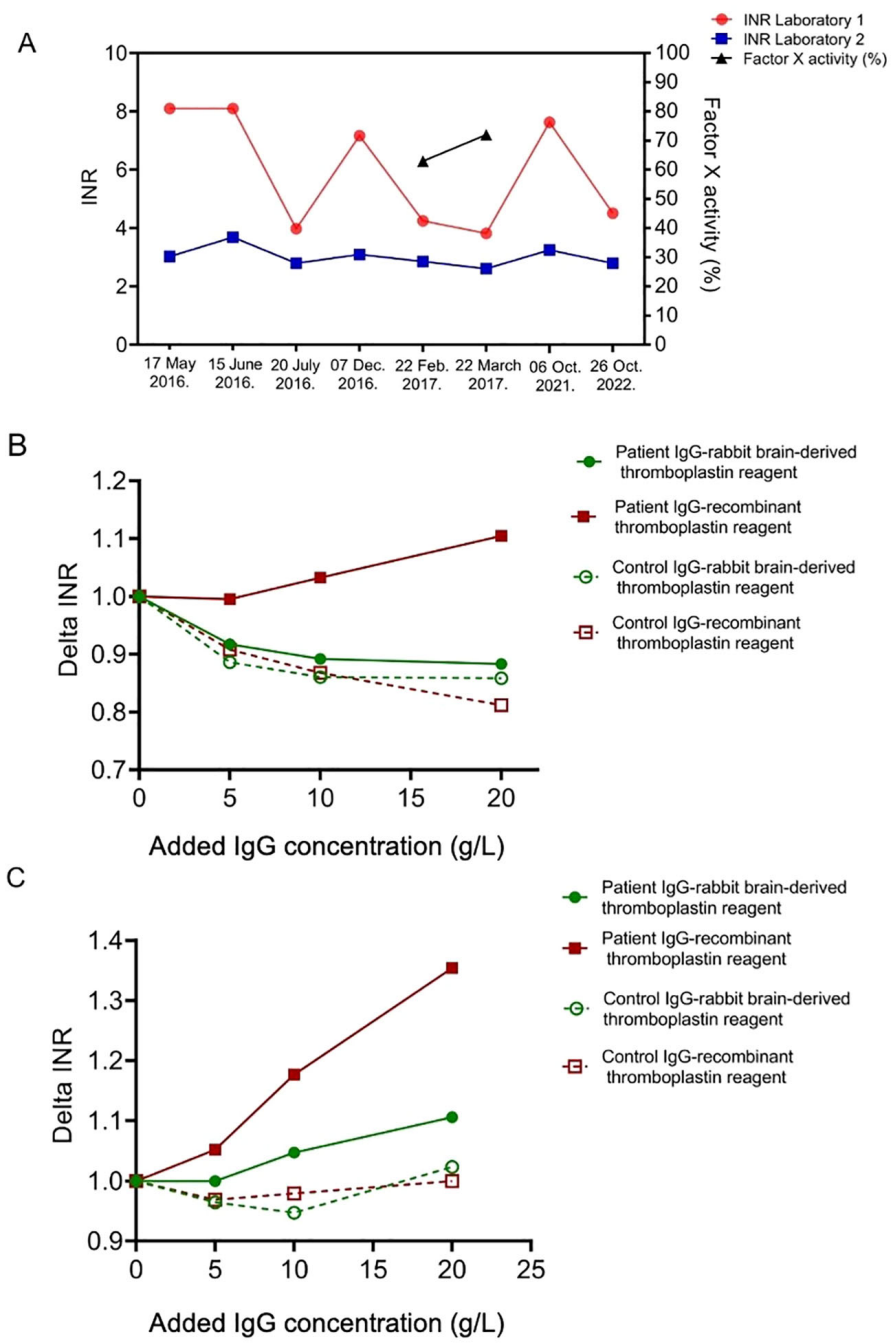
Impact of thromboplastin reagent type on international normalized ratio (INR) measurement in a triple-positive APS patient. **(A)** INR values (left axis) from the plasma sample of a triple-positive APS patient on vitamin K antagonist therapy, measured in two different laboratories (red circles and blue squares) over time. Laboratory 1 had been using recombinant thromboplastin (Innovin, ISI: 0.91, Siemens Healthcare Diagnostics, Marburg, Germany, red circles), while Laboratory 2 used animal-derived, rabbit brain thromboplastin (Dia-PT, ISI: 1.16, Diagon, Budapest, Hungary) to assess INR. Chromogenic factor X activity (%) (black triangles) is plotted on the right axis to assess anticoagulation effectiveness. The corresponding values of factor X activity, Laboratory 1 INR, and Laboratory 2 INR were as follows: 63%, 4.25 and 2.86 (February 22, 2017) and 72%, 3.82 and 2.61 (March 22, 2017), respectively. **(B)** Changes in INR (Delta INR) after adding increasing concentrations of purified patient IgG (solid symbols) or control IgG (open symbols) to aPL-negative control plasma, measured using either rabbit brain-derived thromboplastin (green circles) or recombinant thromboplastin (red squares). **(C)** Changes in INR (Delta INR) after adding increasing concentrations of purified patient IgG (solid symbols) or control IgG (open symbols) to plasma with therapeutic INR (3.5).

The patient was first seen in our adult hematology clinic at the age of 18 while on stable vitamin K antagonist therapy (acenocoumarol 5 mg daily). His INR was 3.72 at that visit, measured using rabbit brain-derived thromboplastin (Dia-PT, ISI: 1.16, Diagon, Budapest, Hungary). However, subsequent INR values showed considerable variability depending on the laboratory and thromboplastin reagent used (**Figure 1A**). To investigate this discrepancy, we performed parallel INR and chromogenic factor X (CFXa) measurements on two separate occasions, using an amidolytic method (Siemens Healthcare Diagnostics, Marburg, Germany, reference range: 70-120%), based on previous literature (18). CFXa results—reflecting the pharmacodynamic effect of VKA—aligned more closely with INR values obtained using rabbit brain-derived thromboplastin. In contrast, INR measurements with recombinant thromboplastin reagent (Innovin, ISI: 0.91, Siemens Healthcare Diagnostics, Marburg, Germany) were consistently higher and not supported by the moderately reduced FXa levels. This discrepancy was not attributed to INR calibration errors. The different INR values obtained were reproducible using the same coagulometer (BCS XP, Siemens) and the same plasma sample but different thromboplastin reagents (data not shown). This suggested that the recombinant reagent might be more susceptible to interference from antiphospholipid antibodies, leading us to further test the impact of patient-derived IgG on INR measurements with both types of thromboplastins.

Repeat thrombophilia testing at the regional center (University of Debrecen, Hungary) showed persistently high INR values (>8), a prolonged APTT of 145 seconds, and an APTT-LA of 119 seconds. Additional findings included an antithrombin activity of 73%, anti-beta 2 glycoprotein 1 (anti-B2GPI) IgG levels exceeding 6100 U/ml, and anticardiolipin IgG levels surpassing 2024 U/ml. After 12 weeks, repeated antiphospholipid antibody testing confirmed extremely high APA titers, leading to a definitive diagnosis of triple-positive APS, associated with a high risk of thromboembolism. Further etiological workup revealed a congenital IgA deficiency.

INR values measured with the recombinant thromboplastin reagent were influenced in a concentration-dependent manner when purified IgG from the patient was added to aPL-negative control plasma at varying concentrations, while this effect was not observed when INR values were obtained using rabbit brain-derived thromboplastin (**Figure 1B**). As a control, purified IgG from aPL-negative individuals was tested, showing no significant impact on INR measurements. The purified patient IgG, in addition to the IgG concentration already present in the patient’s plasma, significantly prolonged the INR of the VKA-anticoagulated pooled plasma measured with recombinant thromboplastin reagent —an effect that was negligible when using rabbit brain-derived thromboplastin (**Figure 1C**). This result is of particular relevance, as FX activity did not reach the expected therapeutic range in the patient, it was therefore considered that the INR obtained by rabbit brain-derived thromboplastin may also have been affected to some extent by lupus anticoagulant interference. However, based on the above-described experiments, the extent of this interference was not considerable. Of note, prolongation of INR by the purified IgG of the patient in VKA-anticoagulated pooled plasma was particularly pronounced in therapeutic or higher INR ranges. In contrast, no such phenomenon was observed when using IgG from aPL-negative controls.

The patient’s anticoagulation therapy with vitamin K antagonists was closely monitored in a 10-year follow-up period. Anticoagulation adjustments were based on INR values from the local laboratory. INR was monitored every three months at our outpatient clinic, and values remained consistently within the therapeutic range (2.0-3.0). This approach was effective in preventing secondary TE events, as over a 10-year follow-up period, no thromboembolic or hemorrhagic complications were observed.

## Discussion

The clinical significance of accurate INR monitoring in triple-positive APS patients is substantial, given their elevated risk of thromboembolic (TE) events. The primary objective in managing such patients is the prevention of secondary TE, typically achieved through VKA therapy. While direct oral anticoagulants (DOACs) have been explored, findings from the TRAPS study and subsequent follow-ups suggest that rivaroxaban is inadequate for oral anticoagulant therapy (OAT) in this high-risk subgroup (14, 19). Current evidence supports the continued use of VKAs, provided that INR monitoring is precise and unaffected by APA interference.

Commercially available thromboplastin reagents vary in their composition and sensitivity to lupus anticoagulant (LA) interference. A multinational study examined nine thromboplastin reagents, including human recombinant, human placenta-derived, and rabbit brain-derived formulations (16). The objective was to distinguish true LA-induced INR variability from calibration-related artifacts and to identify optimal reagents for OAT monitoring. Key limitations of previous investigations, such as small sample sizes and inconsistent calibration methods, were addressed in this study by implementing centralized sample processing and uniform analytical procedures. Findings indicated that among recombinant thromboplastins, only one exhibited significant LA interference, while the majority of reagents, including recombinant thromboplastin reagents showed minimal or no susceptibility. This underscores the importance of reagent selection, as INR values measured using thromboplastins with instrument-specific International Sensitivity Index (ISI) calibration were deemed reliable. However, newer thromboplastins incorporating relipidated or recombinant tissue factor components may exhibit varying degrees of LA sensitivity. Research by Della Valle et al. demonstrated that INR values measured with recombinant thromboplastins containing synthetic phospholipid components were systematically overestimated in LA-positive patients, particularly when low plasma dilutions (1:3 final dilution) were used. By contrast, reagents incorporating bovine brain-derived thromboplastins at higher plasma dilutions (1:20) yielded more accurate results. This suggests that reagent composition and dilution ratios are critical determinants of INR reliability in APS patients (20).

Consequently, LA interference should be assessed before utilizing such reagents for INR-based OAT monitoring. As an alternative, CFXa measurement may serve as a confirmatory test, albeit with limitations such as cost and prolonged turnaround times (18). It must be noted that in rare cases, a differential diagnosis to consider is lupus anticoagulant-hypoprothrombinemia syndrome (LAHS), a condition characterized by lupus anticoagulant positivity with acquired hypoprothrombinemia, often due to anti-prothrombin antibodies. LAHS can present with markedly prolonged PT/INR and is associated with both thrombotic and severe bleeding complications, making it important to recognize in anticoagulated APS patients with unexpected INR elevations (21).

In APS patients with prolonged baseline INR values prior to warfarin initiation or recurrent TE despite therapeutic INR ranges, CFXa measurement may be indispensable. The utility of CFXa in these clinical situations has been demonstrated by independent groups (18, 22–24). Some experts advocate for dual INR and CFXa monitoring during treatment stabilization, a concept referred to as CFXa-calibrated INR targeting. Supporting this approach, an Italian working group compared INR values obtained using two different thromboplastins across 16 LA-positive and 11 LA-negative patients (25). Notably, they concluded that INR values exceeding 4.0 were disproportionately observed in LA-positive samples, reinforcing the need for supplementary CFXa testing in such cases. CFXa typically shows a strong inverse correlation with INR values between 2.0 and 3.0, which supports its utility in assessing anticoagulation intensity within this therapeutic window. However, it is important to note that the interpretation of FX activity measurements becomes increasingly uncertain at supratherapeutic INR levels, particularly above 3.5. At these high INR values, the correlation between FX activity and anticoagulation intensity weakens, limiting the clinical utility of CFXa for monitoring purposes. Efthymiou et al. reported that FX activity may overestimate anticoagulant effect in such settings (18), while Rosborough et al. highlighted the reduced accuracy in patients on warfarin initiation compared with chronic warfarin administration (23). These findings suggest that FX activity results should be interpreted with caution during the initiation of warfarin treatment and when INR exceeds the upper therapeutic threshold.

Compared to prior studies that assessed thromboplastin reagent sensitivity in APS patients, our case report provides a unique combination of clinical, laboratory, and mechanistic insights. While earlier investigations primarily compared INR variability across different reagents in broader APS cohorts, our study is distinguished by its focus on a single, well-characterized triple-positive patient with extremely high APA titers and recurrent thromboembolism. Importantly, we employed patient-derived purified IgG to demonstrate a concentration-dependent interference with recombinant thromboplastin reagents—an approach rarely utilized in prior reports. Given the limited literature addressing the impact of thromboplastin reagent variability in antiphospholipid syndrome, particularly in triple-positive patients, our case report provides timely and clinically relevant insights that may assist both clinical laboratories and physicians in optimizing anticoagulation monitoring strategies. In a most recent study, pooled normal plasma and control plasma from patients on VKA (without LA) were incubated with monoclonal and isolated patient immunoglobulin G antiprothrombin and anti–beta-2-glycoprotein I antibodies that express LA activity. INR was determined before and after addition using 3 laboratory assays (Owren STA-Hepato Prest, Quick STA-NeoPTimal, and Quick STA-Neoplastine R) and 1 point-of-care test device (CoaguChek Pro II) (26). In line with our findings, the authors concluded that INR reagents that utilized recombinant human thromboplastin were more sensitive to the presence of monoclonal and patient-derived antibodies with LA activity, consequently, APS patients positive for LA were suggested to be monitored using tissue-derived thromboplastin reagents.

It should be emphasized that determining the true INR value in patients with triple-positive antiphospholipid syndrome is not merely a technical issue but a clinical imperative. Inaccurate INR readings—particularly overestimations due to reagent sensitivity—can lead to inappropriate dose adjustments, resulting in under-anticoagulation with risk of thromboembolic recurrence or over-anticoagulation with bleeding complications. Given the high thrombotic risk in these patients and the limitations of DOACs in this population, the reliability of INR measurement directly impacts patient safety and long-term outcomes. Therefore, individualized INR monitoring strategies, guided by thromboplastin reagent sensitivity and, when necessary, confirmed with CFXa assays, are essential to ensure accurate anticoagulation assessment and therapeutic efficacy. Our findings, while derived from a single-patient case study, offer a valuable starting point for future investigations and encourage methodological refinements in anticoagulation monitoring for APS patients.

## Conclusions

This report highlights the importance of thromboplastin reagent selection in the INR-based monitoring of high-risk, triple-positive APS patients on VKA therapy. INR values obtained with low-plasma-diluted recombinant thromboplastins were significantly elevated compared to those measured with higher-plasma-diluted rabbit brain thromboplastins, particularly in the upper INR range. This discrepancy was not observed in aPL-negative controls. As a confirmatory test, CFXa activity measurement correlated more closely with INR values obtained using rabbit brain-derived thromboplastin, supporting its use as a reliable tool in cases of suspected LA interference within the therapeutic INR range. Given the critical implications for patient safety, we recommend prioritizing high-plasma-dilutions with animal-derived, or combined thromboplastin reagents for INR monitoring in triple-positive APS patients receiving VKAs.

## Limitations

While our study provides valuable insights into INR measurement discrepancies in triple-positive APS patients, several limitations should be considered. First, our findings are based on a single high-risk patient, necessitating validation in larger cohorts to confirm the generalizability of our results. Second, variability among commercial thromboplastin reagents underscores the need for standardization in INR monitoring, as reagent composition and ISI calibration may differ across laboratories. Third, due to limited sample availability, FX activity measurement was only performed on two occasions in this study, a limitation that was beyond the control of the investigators. It must be noted that while this test may serve as a relevant confirmatory test within the therapeutic INR range, its routine clinical application is often hindered by high costs, long turnaround times, and limited accessibility in many healthcare settings. Finally, further studies are needed to explore the impact of additional confounding factors, such as coexisting prothrombotic conditions and genetic predispositions, on INR variability in APS patients.

## Data Availability

The raw data supporting the conclusions of this article will be made available by the authors, without undue reservation.
